# New Enantiomers of a Nor-Bisabolane Derivative and Two New Phthalides Produced by the Marine-Derived Fungus *Penicillium chrysogenum* LD-201810

**DOI:** 10.3389/fmicb.2021.727670

**Published:** 2021-08-09

**Authors:** Yan Ge, Wen-Li Tang, Qing-Rong Huang, Mao-Lian Wei, You-Zhi Li, Lin-Lin Jiang, Cheng-Lin Li, Xin Yu, Hong-Wei Zhu, Guo-Zhong Chen, Jian-Long Zhang, Xing-Xiao Zhang

**Affiliations:** ^1^School of Life Sciences, Ludong University, Yantai, China; ^2^Shandong Provincial Key Laboratory of Quality Safty Monitoring and Risk Assessment for Animal Products, Jinan, China; ^3^Shandong Aquaculture Environmental Control Engineering Laboratory, Yantai, China; ^4^Yantai Key Laboratory of Animal Pathogenetic Microbiology and Immunology, Yantai, China; ^5^Department of Oncology, Linyi people’s Hospital, Linyi, China

**Keywords:** marine fungus, *Penicillium chrysogenum*, secondary metabolites, bisabolane derivatives, phthalides, antifungal activity

## Abstract

Marine-derived fungi are a treasure house for the discovery of structurally novel secondary metabolites with potential pharmaceutical value. In this study, a pair of new nor-bisabolane derivative enantiomers (±)−1 and two new phthalides (4 and 5), as well as four known metabolites, were isolated from the culture filtrate of the marine algal-derived endophytic fungus *Penicillium chrysogenum* LD-201810. Their structures were established by detailed interpretation of spectroscopic data (1D/2D NMR and ESI-MS). The optical resolution of compound (±)−1 by chiral HPLC successfully afforded individual enantiomers (+)−1 and (−)−1, and their absolute configurations were determined by TDDFT-ECD calculations. Compound (±)−1 represents the first example of bisabolane analogs with a methylsulfinyl substituent group, which is rare in natural products. All of the isolated compounds 1–7 were evaluated for their cytotoxic activity against A549, BT-549, HeLa, HepG2, MCF-7, and THP-1 cell lines, as well as for antifungal activity against four plant pathogenetic fungi (*Alternaria solani*, *Botrytis cinerea*, *Fusarium oxysporum*, and *Valsa mali*). Compound 2, a bisabolane-type sesquiterpenoid, was shown to possess excellent activity for control of *B. cinerea* with half-maximal inhibitory concentration (IC_50_) of 13.6 μg/mL, whereas the remaining investigated compounds showed either weak or no cytotoxic/antifungal activity in this study.

## Introduction

Marine-derived fungi that inhabit the marine environment possess the unique metabolic pathways to produce a great diversity of bioactive secondary metabolites, which play an important role in agrochemical and pharmaceutical industries (Xu et al., 2020; Zhang et al., 2020). It is well-known that a large number of new marine natural products have been discovered and reported every year (Carroll et al., 2021). Mining natural products with novel structures and remarkable bioactivities from marine-derived fungi is still a research hotspot.

**FIGURE 1:**
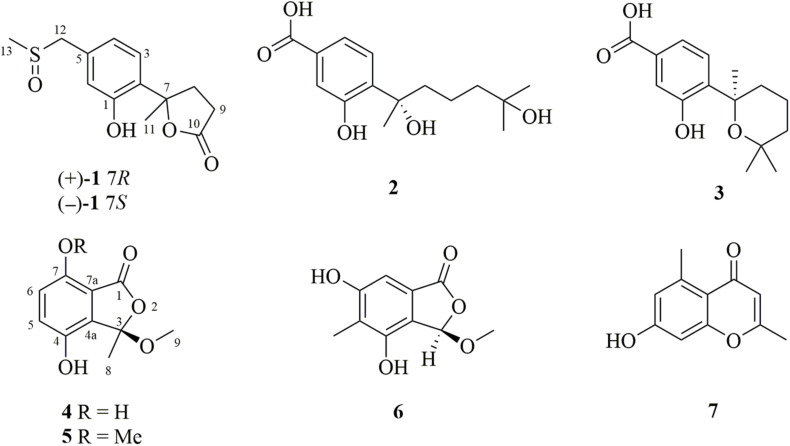
Chemical structures of the isolated compounds 1–7 from *P. chrysogenum* LD-201810.

Filamentous fungi belonging to the genus *Penicillum* are important and untapped producers of structurally diverse metabolites (Bai et al., 2019). *Penicillum* from marine environment have gained particular attention, not only due to their unusual chemical skeletons but also their significant bioactivities with pharmaceutical potential (Zhang et al., 2020). In our continuing study on bioactive metabolites of marine-derived fungi, we investigated *Penicillium chrysogenum* LD-201810, a marine alga-associated fungus isolated from the marine red alga *Grateloupia turuturu* (Jiang et al., 2020). Previous solid cultivation of this fungus on rice medium led to the isolation of a new pentaketide derivative, two new hydroxyphenylacetic acid derivatives, as well as the known bisabolane-type sesquiterpenoids and meroterpenoids (Jiang et al., 2020). Motivated by OSMAC (one strain-many compounds) strategy (Zhao et al., 2020), the fungal strain was cultivated on liquid PDB medium. A follow-up examination of this cultivation yielded a pair of new nor-bisabolane derivative enantiomers (±)−1, two previously reported bisabolenes (2 and 3), and two new phthalides (4 and 5) (Figure 1). The aromatic bisabolenes are a rarely found family of sesquiterpenes. Mulholland et al. firstly reported a new trisnor-bisabolane sesquiterpene boivinianin A (Mulholland et al., 2006). Then Li et al. (2015) reported the second occurrence of a new nor-bisabolane derivative, 1-hydroxyboivinianin A. Herein we reported the first example of nor-bisabolane analogs with a methylsulfinyl substituent group, which is rare in natural products. Moreover, the structure elucidation of the new phthalides (4 and 5), as well as the cytotoxicity and antifungal activity of the isolated compounds, are also described.

## Materials and Methods

### General Experimental Procedures

The UV and optical rotations data were obtained on a Shimadzu UV-2700 spectrometer (Shimadzu Co., Ltd., Kyoto, Japan) and Jasco P-1020 automatic polarimeter (JASCO, Tokyo, Japan), respectively. ^1^H (500 MHz), ^13^C (125 MHz), and 2D NMR spectra were measured on an Agilent DD2 spectrometer (Agilent Technologies, Waldbronn, Germany). The mass spectra (ESI-MS) were measured under the positive and negative ion modes by a Waters Xevo G2-XS QTof mass spectrometer (Waters, Milford, MA, United States). Column chromatography was performed on silica gel (100–200 and 200–300 mesh, Qingdao Marine Chemical Inc., Qingdao, China), Lobar LiChroprep RP-18 (40–60 μm, Merck, Darmstadt, Germany), and on Sephadex LH-20 (Merck). Preparative TLC plates precoated with silica gel GF_254_ were purchased from Qingdao Marine Chemical Industry Company.

### Fungal Material

The producing fungal strain *P. chrysogenum* LD-201810 was isolated from *Grateloupia turuturu*, a marine red alga which collected in Qingdao coastal zone. The gene sequencing in the ITS region of the rDNA (GenBank no. MT075873) was applied to identify the fungus (Jiang et al., 2020). To identify the phylogenetic location of this fungus, phylogenetic trees were constructed based on the ITS region sequences using maximum likelihood (ML) method, and the bootstrap support was calculated using 1,000 replicates. The fungus has been deposited in School of Life Sciences, Ludong University, Yantai, China.

### Fermentation, Extraction, and Isolation

The selected fungal strain *P. chrysogenum* LD-201810 was cultured on potato dextrose agar (PDA) medium (Solarbio Life Sciences, Beijing) at 28°C. After 5 days, the agar blocks were cut into small pieces (0.5 × 0.5 cm), and then inoculated to 100 erlenmeyer flasks containing the liquid potato dextrose broth (PDB) medium (Solarbio Life Sciences) under static conditions at room temperature for 30 days. The culture filtrate was collected, combined, and extracted with equivoluminal EtOAc (30 L) for three times. The organic phase was subsequently dried under reduced pressure to afford 12.6 g of crude extract. Then the detailed separation process was as follows: (i) The crude extract was subjected to open silica gel column chromatography (CC) (100−200 mesh), eluted with a mixed petroleum ether (PE)−EtOAc gradient system (30:1, 10:1, 5:1, 2:1, 1:1, and 0:1) to yield six fractions (Fr. 1 to Fr. 6); (ii) Fr. 4 (2.5 g, eluted with PE−EtOAc 2:1, v/v) was re-fractionated by reversed-phase CC over Lobar LiChroprep RP-18 with a MeOH−H_2_O gradient system (from 10% MeOH−H_2_O to 100% MeOH, v/v) to give nine subfractions (Fr. 4.1 to Fr. 4.9); (iii) Fr. 4.3 (0.5 g, eluted with 30% MeOH−H_2_O, v/v) was separated by preparative thin layer chromatography [prep.-TLC, 20 × 20 cm; developing solvents: dichloromethane (DCM)−methanol (MeOH), 20:1, v/v] to furnish the new compounds 4 (4.5 mg) and 5 (8.0 mg); (iv) Fr. 4.4 (0.8 g, eluted with 40% MeOH−H_2_O, v/v) was also separated by prep.-TLC (developing solvents: DCM−MeOH, 20:1, v/v) to obtain compound 6 (12.0 mg); (v) Fr. 4.6 (0.6 g, eluted with 60% MeOH−H_2_O, v/v) was repeatedly subjected to Sephadex LH-20 (MeOH) to give compound 7 (20.5 mg); (vi) Fr. 5 (1.9 g, eluted with PE−EtOAc 1:1, v/v) was subjected to Sephadex LH-20 (MeOH) to yield five subfractions (Fr. 5.1 to Fr. 5.5); (vii) Fr. 5.2 (0.8 g) was applied to silica gel CC (200–300 mesh; DCM−MeOH 20:1, v/v) to yield compounds 2 (9.6 mg) and 3 (31.8 mg); (viii) Fr. 5.3 (0.4 g) was purified by prep.-TLC (developing solvents: DCM−MeOH, 10:1, v/v) to give the racemic compound 1 (7.0 mg). Compound 1 was well resolved into the pure enantiomers (+)−1 (3.0 mg, *t*_R_ = 16.3 min) and (−)−1 (2.8 mg, *t*_R_ = 18.3 min) by HPLC using a (*R*,*R*) Whelk-O1 chiral column (10 μm; 4.6 × 250 mm; *n*-hexane-ethanol eluent 7:3, v/v; 1.0 mL/min).

Methylsulfinyl-1-hydroxyboivinianin A (±1): white amorphous powder; UV (MeOH) λ_max_ (log ε) 200 (2.65), 282 (1.52) nm; ^1^H and ^13^C NMR data were assigned and listed in Table 1; (−)-HR-ESI-MS *m/z* 267.0680 [M − H]^–^ (calcd for C_13_H_15_O_4_S, 267.0691).

**TABLE 1:** ^1^H (500 MHz) and ^13^C NMR (125 MHz) data of the new compounds 1, 4, and 5.

**No.**	**Compound (±)-1^a^**		**No.**	**Compound 4^b^**		**Compound 5^b^**	
	**δ_H_ (mult, *J* in Hz)**	**δ_C_, type**		**δ_H_ (mult, *J* in Hz)**	**δ_C_, type**	**δ_H_ (mult, *J* in Hz)**	**δ_C_, type**
1		155.0, C	1		166.2, C		165.5, C
2		132.0, C	3		107.2, C		107.1, C
3	7.33 (d, 8.4)	126.5, CH	4		145.2, C		146.5, C
4	6.83 (d, 8.4)	122.3, CH	4a		131.3, C		132.7, C
5		132.3, C	5	7.01 (d, 8.7)	124.3, CH	7.14 (d, 8.8)	123.8, CH
6	6.82 (s)	118.9, CH	6	6.84 (d, 8.7)	119.7, CH	7.06 (d, 8.8)	115.6, CH
7		88.6, C	7		149.7, C		150.9, C
8	2.67 (m) 2.49 (m)	35.1, CH_2_	7a		113.1, C		114.8, C
9	2.65 (m) 2.45 (m)	29.7, CH_2_	8	1.73 (s)	24.2, CH_3_	1.75 (s)	24.1, CH_3_
10		179.6, C	9	2.93 (s)	51.0, CH_3_	2.93 (s)	51.1, CH_3_
11	1.76 (s)	26.7, CH_3_	10	–	–	3.80 (s)	56.3, CH_3_
12	4.07 (d, 13.0) 3.94 (d, 13.0)	59.7, CH_2_					
13	2.58 (s)	37.4, CH_3_					

*^*a*^Measured in CD_3_OD.*

*^*b*^Measured in DMSO-*d*_6_.*

(+)−1: [α]^25^_D_ + 23.8° (c 0.05, MeOH); ECD (0.125 mg/mL, MeOH) λ_max_ (Δε) 220 (+ 1.54) nm.

(−)−1: [α]^25^_D_ −22.2° (c 0.04, MeOH); ECD (0.125 mg/mL, MeOH) λ_max_ (Δε) 218 (−1.52) nm.

Chrysoalide A (4): white amorphous powder; [α]^25^_D_ + 31.5° (c 0.03, MeOH); UV (MeOH) λ_max_ (log ε) 216 (2.58), 239 (2.03), 330 (1.96) nm; ^1^H and ^13^C NMR data were assigned and listed in Table 1; (−)-HRESIMS *m/z* 209.0431 [M − H]^–^ (calcd for C_1__0_H_9_O_5_, 209.0450).

Chrysoalide B (5): white amorphous powder; [α]^25^_D_ + 15.8° (c 0.03, MeOH); UV (MeOH) λ_max_ (log ε) 215 (2.13), 237 (1.63), 329 (1.50) nm; ^1^H and ^13^C NMR data were assigned and listed in Table 1; (−)-HRESIMS *m/z* 223.0584 [M − H]^–^ (calcd for C_11_H_11_O_5_, 223.0606).

### Computational Section

The conformational search was performed by the molecular mechanics with MM + method in HyperChem 8.0 software. Next, the geometries were optimized at B3LYP/6-31G(d) level with Gaussian 09 software to afford the energy-minimized conformers (Frisch et al., 2013). The optimized conformers were subjected to TD-DFT ECD calculations at PBE0/TZVP, CAM-B3LYP/TZVP, and BH&HLYP/TZVP level. The solvent effects (MeCN) were evaluated at the same DFT level with the SCRF/PCM method.

### Cytotoxic Assay

Cytotoxicity of compounds 1−7 toward A549, BT-549, HeLa, HepG2, MCF-7, and THP-1 cell lines was tested by the Cell Counting Kit-8 (CCK-8) method (Yuan et al., 2020). All of the cell lines were purchased from the Chinese Academy of Sciences Committee on Type Culture Collection Cell Bank (Shanghai, China). The six cell lines (3.0 × 10^4^ cells per well) were initially inoculated into 96-well plates for 24 h. Subsequently, the cells were exposed to various concentrations of tested compounds (0, 5, 10, 20, 40, 80, and 100 μg/mL). With the treatment of 24, 48, and 72 h, 10 μL of 5 g/L CCK-8 solution (CCK-8 Cell Proliferation and Cytotoxicity Assay Kit, #CA1210, Solarbio, Beijing, China) was applied to each well and the cells were cultured for 1.5 h at 37°C. Absorbance data were obtained with a microplate spectrophotometer reader (Multiskan GO, Thermo Fisher Scientific, Waltham, MA, United States) at 490 nm.

### Antifungal Assay

The antifungal activities against four phytopathogenic fungi (*Alternaria solani, Botrytis cinerea, Fusarium oxysporum*, and *Valsa mali*) were evaluated in 96-well microtiter plates using a modified broth microdilution method (Shi et al., 2017). Carbendazim was used as a positive control. The tested compounds were added to autoclaved PDA medium to a final concentration of 3.12, 6.25, 12.5, 25, and 50 μg/mL, while 95% ethanol was treated as black control. The blocks (about 5 mm diameter) from four phytopathogenic fungi were cultured in the center of plates at 20°C. Colony diameters were measured with the cross method. The mycelial growth inhibition rate was calculated as follows, while IC_50_ values were obtained by the logarithm method.

The mycelial growth inhibition rate = (control colony diameter – treatment colony diameter)/(control colony diameter – 5) × 100%

## Results and Discussion

### Identification of the Producing Strain

**FIGURE 2:**
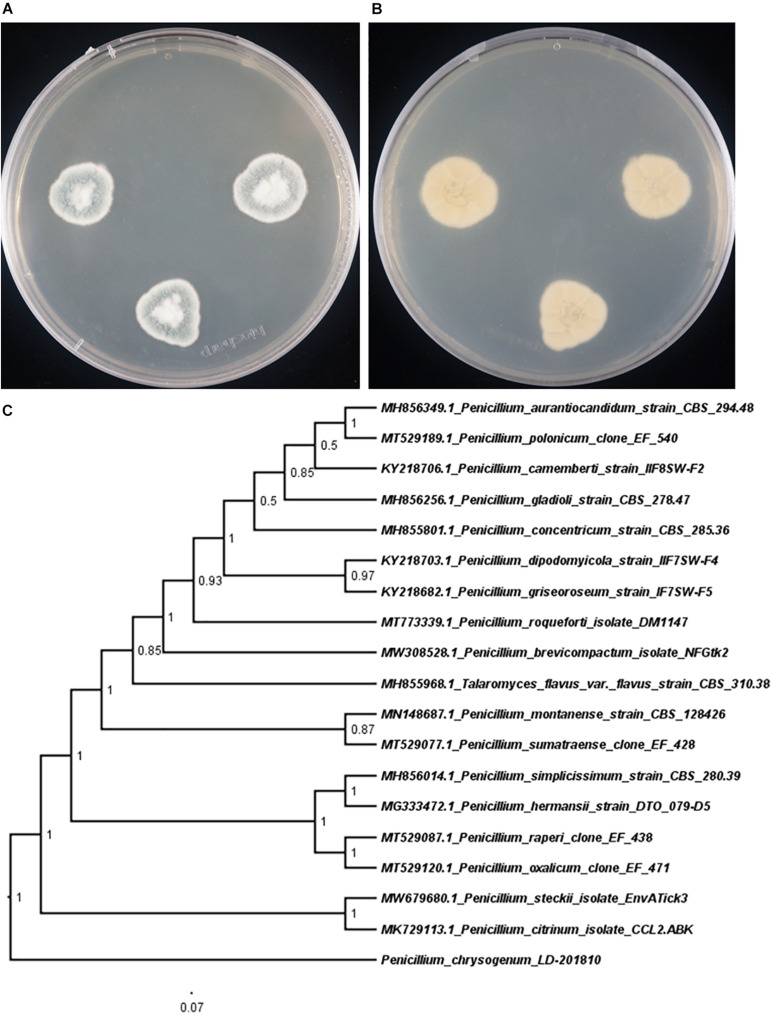
Morphology of *P. chrysogenum* LD-201810 on PDA medium (**A**, front view; **B** reverse view). **(C)** Neighbor-joining tree based on ITS nucleotide sequences.

To clarify the evolutionary position of the producing strain LD-201810, we performed phylogenetic analysis based on its ITS sequence, together with those from other *Penicillium* species. Results indicated that the strain LD-201810 located at the basal position of the whole tree with high confidence (100%, Figure 2). The result demonstrated that *Penicillium chrysogenum* LD-201810 belongs to the *Penicillium* genus.

### Structural Elucidation

**FIGURE 3:**
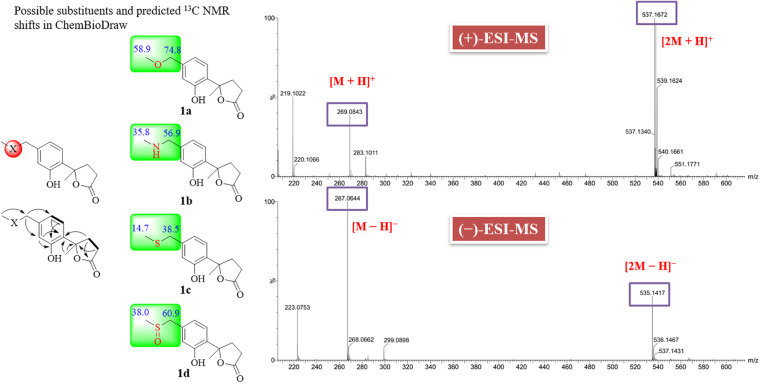
COSY, key HMBC correlations and possible substituents of compound (±)−1.

Compound (±)−1 was a white amorphous powder (MeOH), and its molecular formula was determined to be C_13_H_16_O_4_S by negative-mode HR-ESI-MS (*m/z* 267.0680 [M − H]^–^, calcd 267.0691). The ^1^H NMR data for 1 (Table 1) clearly revealed signals of two methyl singlets at δ_H_ 1.76 (s, H_3_-11) and 2.58 (s, H_3_-13), two sets of methylene multiplets at δ_H_ 2.67 (m, H-8α), 2.65 (m, H-9α), 2.49 (m, H-8β), and 2.45 (m, H-9β), a pair of methylene doublets at δ_H_ 4.07 (d, *J* = 13.0 Hz, H-12α) and 3.94 (d, *J* = 13.0 Hz, H-12β), and three aromatic protons at δ_H_ 7.33 (d, *J* = 8.4 Hz, H-3), 6.83 (d, *J* = 8.4 Hz, H-4), and 6.82 (s, H-6). The ^13^C NMR data identified 13 carbon signals that were highly resolved, categorized as two methyls, three methylenes, three sp^2^ methines, and five quaternary carbons including three sp^2^, one oxygenated sp^3^, and one carbonyl carbon at δ_C_ 179.6 (C-10). Detailed analysis of the 1D and 2D NMR (Figure 3) spectra of 1 indicated that they were similar to those of 1-hydroxyboivinianin A, a trisnor-bisabolane derivative identified from the culture of a deep-sea sediment-derived fungus *Penicillium aculeatum* SD-321 (Li et al., 2015). By comparison of the NMR data of 1-hydroxyboivinianin A with those of 1, the main differences in 1 were the presence of an additional methylene group at δ_C_ 59.7 (C-12) and a distinctive methyl at δ_C_ 37.4 (C-13). The downfield chemical shifts of 13-CH_3_ (δ_H__/C_ 2.58/37.4) and 12-CH_2_ (δ_C_ 59.7) were ascribed to that bearing a heteroatom between them. Initially, the common-observed heteroatoms, such as oxygen (1a), nitrogen (1b), and sulfur (1c) atoms, were assumed between C-13 and C-12. However, the predicted ^13^C NMR shifts in ChemBioDraw didn’t match well with that for measured data (Figure 3). Furthermore, combined with positive-mode ESI-MS (*m/z* 269.0843 [M + H]^+^ and 537.1672 [2M + H]^+^) and negative-mode ESI-MS (*m/z* 267.0644 [M − H]^–^ and 535.1417 [2M − H]^–^), the molecular weight of 1 was determined as 268. In view of its molecular weight, a remaining S and O atom could be accounted for by inserting the S = O group between C-13 and C-12 to form a methylsulfinyl substituent. The predicted data for 1d were in good agreement with the authentic data. Moreover, Fu et alreported a series of synthetic compounds with a methylsulfinyl group (Fu et al., 2020). The chemical shifts of C-12 and C-13 in 1 were accordant with those of known compounds, which further confirmed the presence of such a rare substituent in natural products. On the basis of the above discussion, the structure of compound 1 was determined as methylsulfinyl-1-hydroxyboivinianin A.

**FIGURE 4:**
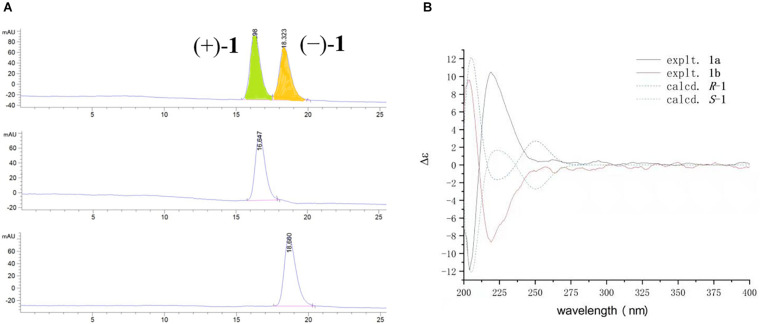
**(A)** Chromatogram of the chiral HPLC analysis of 1. **(B)** Experimental and calculated ECD spectra of (±)−1 in MeOH.

Compound 1 had only one chiral center at C-7. The zero specific rotation value and baseline ECD curve indicated its racemic nature (Meng et al., 2016). Subsequent chiral HPLC analysis of (±)−1 successfully led to the separation of the two individual enantiomers (+)−1 and (−)−1 with a ratio of approximately 1:1, which exhibited opposite optical rotations (Figure 4A). To determine the absolute configurations of (+)−1 and (−)−1, their ECD spectra were measured in MeOH and simulated by the time-dependent density function theory (TD-DFT) method. The experimental ECD spectrum of (+)−1 showed a positive (+220 nm) Cotton effect, whereas the experimental (−)−1 showed an almost mirror image ECD curve (Figure 4B). The calculated ECD curves of 7*R* and 7*S* matched the experimental ECD curves of (+)−1 and (−)−1, thus the absolute configurations of (+)−1 and (−)−1 were proposed as 7*R* and 7*S*, respectively.

**FIGURE 5:**
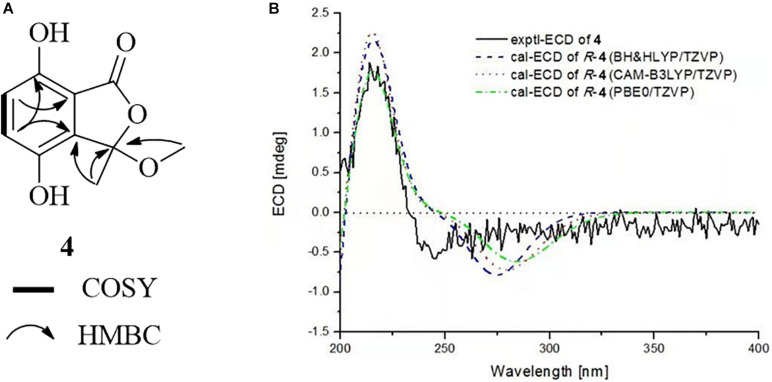
**(A)** COSY and HMBC correlations of **4**. **(B)** Experimental and calculated ECD spectra of **4** in MeOH.

Chrysoalide A (4), a white amorphous powder, was shown to possess a molecular formula of C_1__0_H_1__0_O_5_ by its HR-ESI-MS (*m/z* 209.0431 [M − H]^–^, calcd 209.0450). Its UV spectrum showed absorption peaks at 216, 239, and 330 nm, indicating the presence of a conjugated carbonyl chromophore (Phainuphong et al., 2018; Saetang et al., 2021). The ^1^H NMR data for 4 (Table 1) showed signals of one methyl singlet at δ_H_ 1.73 (s, H_3_-8), one methoxy singlet at δ_H_ 2.93 (s, H_3_-9), and a pair of intercoupling aromatic protons at δ_H_ 7.01 (d, *J* = 8.7 Hz, H-5) and 6.84 (d, *J* = 8.7 Hz, H-6), which can be easily deduced the presence of a 1,2,3,4-tetrasubstituted benzene group. The ^13^C NMR spectrum (Table 1) displayed signals for two methyls at δ_C_ 24.2 (C-8) and 51.0 (C-9), two sp^2^ methines at δ_C_ 124.3 (C-5) and 119.7 (C-6), five quaternary carbons, and one ester carbonyl at δ_C_ 166.2 (C-1). Detailed analysis of 2D NMR data established the planar structure of 4 (Figure 5A). Moreover, the experimental ECD spectrum of 4 displayed a similar shape of curves and Cotton effects to those of the calculated ECD spectrum of the *R*-configuration (Figure 5B), which established the absolute configuration of C-3 to be *R*.

**FIGURE 6:**
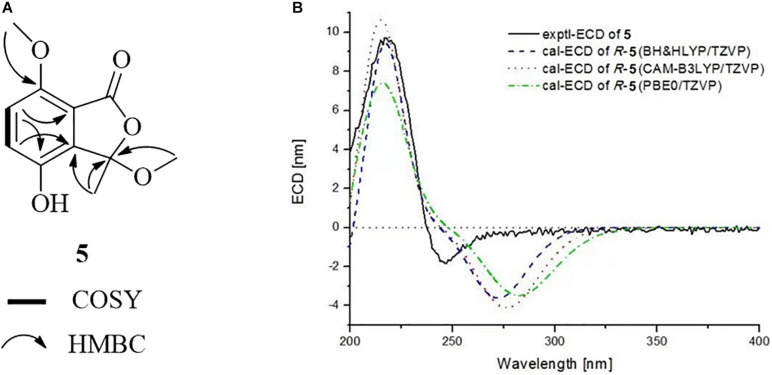
**(A)** COSY and HMBC correlations of **5**. **(B)** Experimental and calculated ECD spectra of **5** in MeOH.

Chrysoalide B (5) was also obtained as a white amorphous powder with a molecular formula of C_11_H_1__2_O_5_ as determined by HR-ESI-MS. The molecular weight of 5 was more than that of 4 by 14 units (CH_2_). The 1D and 2D NMR spectra of 5 (Table 1 and Figure 6A) suggested that it resembled 4 structurally, but possessed an additional methoxy group at δ_H__/C_ 3.80/56.3. The extra methoxy group was shown to be linked to C-7, as evidenced from the HMBC correlation from 10-CH_3_ to C-7. Compound 5 was elucidated as a methoxy derivative of 4. The absolute configuration of 5 was considered to be identical with that of 4 by its similar ECD curve, which gave a positive Cotton effect at 220 nm. Comparison of the experimental ECD data with those of calculated spectra further proved the above assignment (Figure 6B).

In addition to the new compounds, two previously reported bisabolane sesquiterpenes 2 and 3, a phthalide derivative 6, and a chromone 7, were also isolated from this fungal strain. Based on detailed spectroscopic analysis as well as by comparisons with literature data, their structures were identified as hydroxysydonic acid (2) (Hamasaki et al., 1978), sydowic acid (3) (Hamasaki et al., 1975), rubralide C (6) (Kimura et al., 2007), and 2,5-dimethyl-7-hydroxychromone (7) (Kashiwada et al., 1984).

### Bioactivity of the Isolated Compounds

All of the isolated compounds were evaluated for their cytotoxicity against six different types of cancer cell lines, A549 (a human lung adenocarcinoma epithelial cell line), BT-549 (a human breast cancer cell line), HeLa (a human cervix carcinoma cell line), HepG2 (a human liver carcinoma cell line), MCF-7 (a human breast adenocarcinoma cell line), and THP-1 (a human monocytic cell line). However, none of them exhibited obvious inhibitory activity at 20 μg/mL (the highest concentration tested, data were shown in Supplementary Table 1).

**FIGURE 7:**
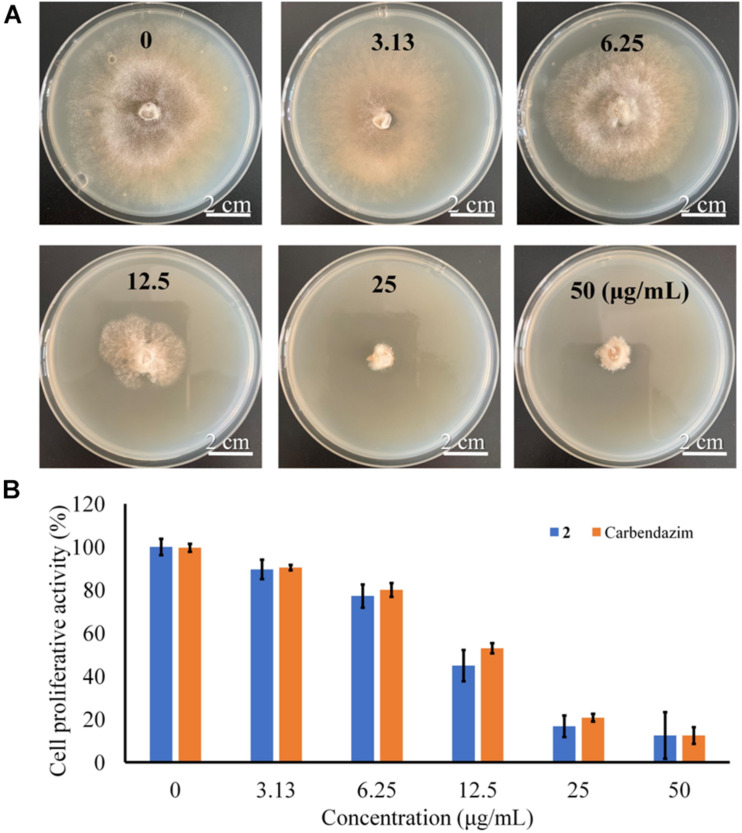
**(A)** Antifungal assay against *B. cinerea* with different concentrations. **(B)** Inhibition of proliferative activity against *B. cinerea* induced by 2.

Previous studies indicated that bisabolane sesquiterpenoids and phthalides possessed promising antimicrobial activity (Li et al., 2015; Saetang et al., 2021). Marine natural products considered to be new sources of lead molecules with agrochemical significance (Oppong-Danquah et al., 2020). To discover new marine fungal agrochemicals, the isolated compounds were evaluated for antifungal activity against several plant pathogenetic fungi (*A. solani*, *B. cinerea*, *F. oxysporum*, and *V. mali*). The bisabolane-type sesquiterpenoid 2 was shown to possess excellent activity for control of *B. cinerea* with an IC_50_ value of 13.6 μg/mL (Figure 7) (compared with the positive control carbendazim, with an IC_50_ value of 19.2 μg/mL), whereas other compounds showed either weak or no activity (Data were shown in Supplementary Table 2). It should be pointed out that limited amounts of these metabolites were obtained, which prevented us to perform more biological experiments. Further study should be particularly focused on more agricultural activities, such as antifeedant and phytotoxic activities, to fully evaluate their agricultural potentials.

## Conclusion

Marine-derived fungi have been proven to be prolific producers of secondary metabolites with potent bioactivities. In this study, chemical investigation of a marine algal-derived endophytic fungus *P. chrysogenum* LD-201810 led to the isolation and identification of a pair of new nor-bisabolane derivative enantiomers (±)−1 and two new phthalides (4 and 5), as well as four known metabolites (2, 3, 6, and 7). Compound (±)−1 represents the first example of bisabolane analogs with a methylsulfinyl substituent group, which is rare in natural products. The aromatic bisabolanes are a rarely found family of sesquiterpenes, and the discovery of (±)−1 added greatly to the diversity of this kind of molecules. The cytotoxic and antifungal activities were evaluated. Hydroxysydonic acid (2), a bisabolane-type sesquiterpenoid, showed strong inhibition against *B. cinerea*, compared with that of the positive control carbendazim. The results indicated that some marine natural products may be regarded as candidate agents of antifungal agrochemicals.

## Data Availability Statement

The original contributions presented in the study are included in the article/Supplementary Material, further inquiries can be directed to the corresponding authors.

## Author Contributions

YG and W-LT: writing-original draft preparation. Q-RH: methodology. M-LW and Y-ZL: investigation. L-LJ and C-LL: formal analysis. XY, H-WZ, and G-ZC: data curation. J-LZ and X-XZ: writing-review and editing. J-LZ: supervision. X-XZ: funding acquisition. All authors have read and agreed to the published version of the manuscript.

## Conflict of Interest

The authors declare that the research was conducted in the absence of any commercial or financial relationships that could be construed as a potential conflict of interest.

## Publisher’s Note

All claims expressed in this article are solely those of the authors and do not necessarily represent those of their affiliated organizations, or those of the publisher, the editors and the reviewers. Any product that may be evaluated in this article, or claim that may be made by its manufacturer, is not guaranteed or endorsed by the publisher.
