# Supplementary material for: New Enantiomers of a Nor-Bisabolane Derivative and Two New Phthalides Produced by the Marine-Derived Fungus Penicillium chrysogenum LD-201810
Source: Front Microbiol. 2021 Aug 9;12:727670. doi: 10.3389/fmicb.2021.727670 (PMC8381153; doi:10.3389/fmicb.2021.727670)
Supplement: Supplementary file 1 [file Data_Sheet_1.docx]

***Supplementary Materials***

**New Enantiomers of a Nor-Bisabolane** **Derivative and Two New Phthalides Produced by the Marine-Derived Fungus** ***Penicillium chrysogenum* LD-201810**

Yan Ge^1,2,4, †^, Wen-Li Tang^2, †^, Qing-Rong Huang^1,3^, Mao-Lian Wei^2^, You-Zhi Li^2^, Lin-Lin Jiang^1,2,4^, Cheng-Lin Li^5^, Xin Yu^1,2,3^, Hong-Wei Zhu^1,2,4^, Guo-Zhong Chen^1,3,4^, Jian-Long Zhang^1,2,4,^ *, and Xing-Xiao Zhang^1,3,4,^ *

^1^ School of Life Sciences, Ludong University, Yantai 264025, China; 851321096@qq.com (Y. G.); ythqr801@163.com (Q.-R. H.); linlinjiang1986@163.com (L.-L. J.); yuxinzghn@163.com (X. Y.); hngwzhu@outlook.com (H.-W. Z.); guozhongchyt@163.com (G.-Z. C)

^2^ Shandong Provincial Key Laboratory of Quality Safty Monitoring and Risk Assessment for Animal Products, Ji'nan 250022, China; 13905310419@163.com (W.-L. T.); 764902337@qq.com (M.-L. W.); liyouzhi2009@126.com (Y.-Z. L.)

^3^ Yantai Key Laboratory of Animal Pathogenetic Microbiology and Immunology, Yantai 264025, China

^4^ Shandong Aquaculture Environmental Control Engineering Laboratory, Yantai 264000, Shandong, China

^5^ Department of oncology, Linyi people's Hospital, Linyi 276000, Shandong, China; 1143397331@qq.com (C.-L. L.)

* Correspondence: zhangjianlong@ldu.edu.cn (J.-L. Z.); zhangxingxiao@ldu.edu.cn (X.-X. Z.); Tel.: +86-535-6681162 (J.-L. Z.); +86-535-6673485 (X.-X. Z.)

^†^ These authors contributed equally to this work.

**Table of Contents**

**Figure S1**. ^1^H NMR (500 MHz, CD_3_OD) spectrum of compound (±)-**1**;

**Figure S2**. ^13^C NMR (125 MHz, CD_3_OD) and DEPT spectra of compound (±)-**1**;

**Figure S3**. HSQC spectrum of compound (±)-**1**;

**Figure S4**. COSY spectrum of compound (±)-**1**;

**Figure S5**. HMBC spectrum of compound (±)-**1**;

**Figure S6**. HRESIMS spectrum of compound (±)-**1**;

**Figure S7**. ^1^H NMR (500 MHz, DMSO-*d*_6_) spectrum of compound **4**;

**Figure S8**. ^13^C NMR (125 MHz, DMSO-*d*_6_) and DEPT spectra of compound **4**;

**Figure S9**. HSQC spectrum of compound **4**;

**Figure S10**. COSY spectrum of compound **4**;

**Figure S11**. HMBC spectrum of compound **4**;

**Figure S12**. HRESIMS spectrum of compound **4**;

**Figure S13**. ^1^H NMR (500 MHz, DMSO-*d*_6_) spectrum of compound **5**;

**Figure S14**. ^13^C NMR (125 MHz, DMSO-*d*_6_) and DEPT spectra of compound **5**;

**Figure S15**. HSQC spectrum of compound **5**;

**Figure S16**. COSY spectrum of compound **5**;

**Figure S17**. HMBC spectrum of compound **5**;

**Figure S18**. HRESIMS spectrum of compound **5**

**Table S1**. Cytotoxicity of compounds **1**–**7** at 20 μg/mL (inhibition ratio, %)

**Table S2**. Antifungal activity of compounds **1**–**7** (IC_50_, μg/mL)

** Figure S1**. ^1^H NMR (500 MHz, CD_3_OD) spectrum of compound (±)-**1**;

** Figure S2**. ^13^C NMR (125 MHz, CD_3_OD) and DEPT spectra of compound (±)-**1**;

** Figure S3**. HSQC spectrum of compound (±)-**1**;

** Figure S4**. COSY spectrum of compound (±)-**1**;

**Figure S5**. HMBC spectrum of compound (±)-**1**;

**
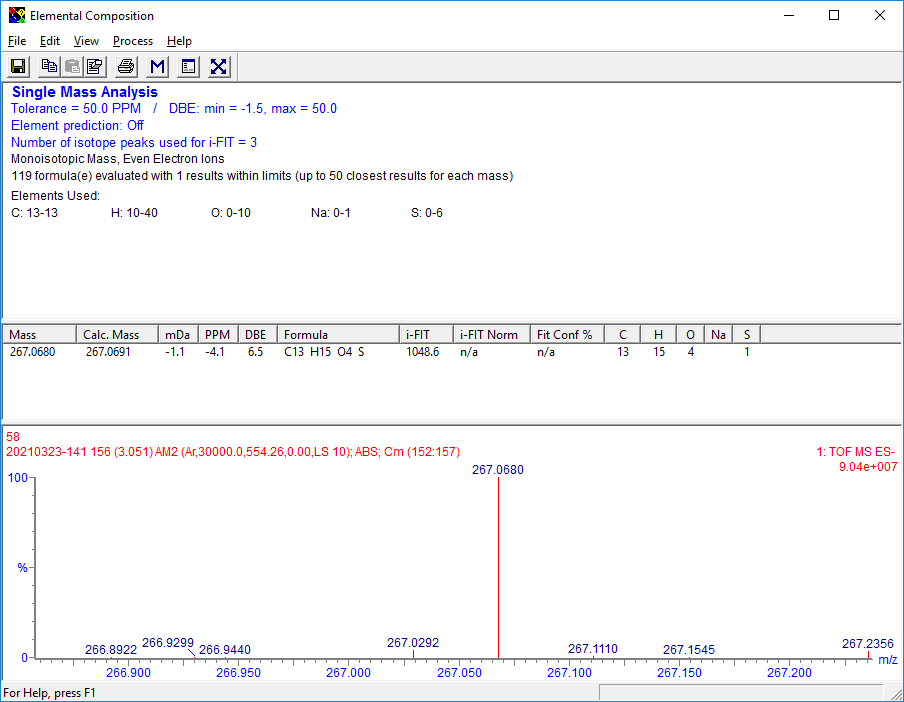
**

**Figure S6**. HRESIMS spectrum of compound (±)-**1**;

**Figure S7**. ^1^H NMR (500 MHz, DMSO-*d*_6_) spectrum of compound **4**;

**Figure S8**. ^13^C NMR (125 MHz, DMSO-*d*_6_) and DEPT spectra of compound **4**;

**Figure S9**. HSQC spectrum of compound **4**;

**Figure S10**. COSY spectrum of compound **4**;

**Figure S11**. HMBC spectrum of compound **4**;

**
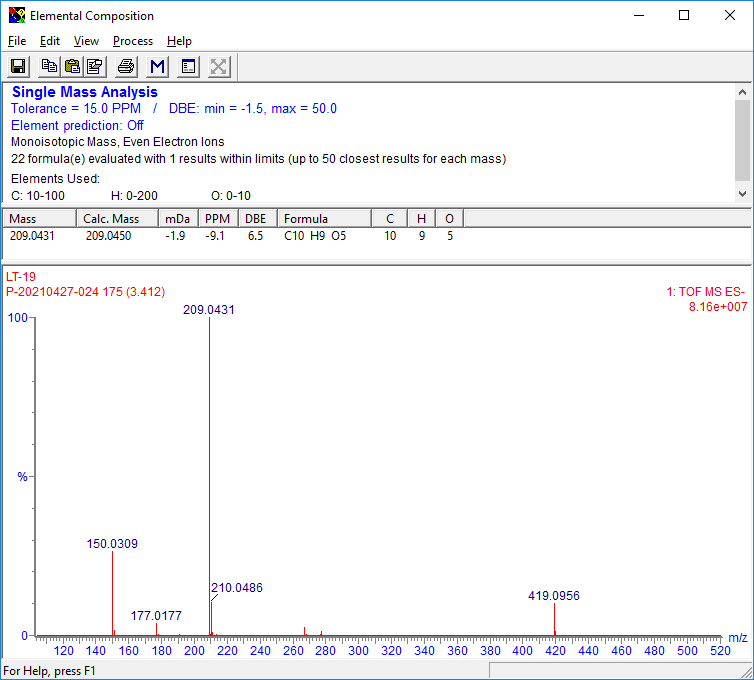
**

**Figure S12**. HRESIMS spectrum of compound **4**;

**Figure S13**. ^1^H NMR (500 MHz, DMSO-*d*_6_) spectrum of compound **5**;

**Figure S14**. ^13^C NMR (125 MHz, DMSO-*d*_6_) and DEPT spectra of compound **5**;

**Figure S15**. HSQC spectrum of compound **5**;

**Figure S16**. COSY spectrum of compound **5**;

**Figure S17**. HMBC spectrum of compound **5**;

**
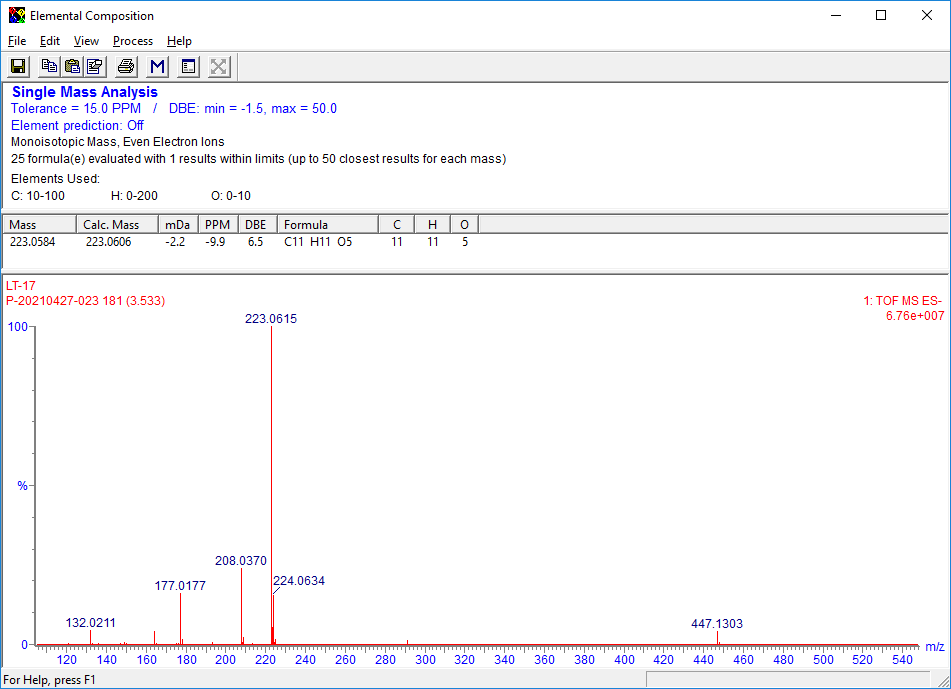
Figure S18**. HRESIMS spectrum of compound **5**

**Table S1.** Cytotoxicity of compounds **1**–**7** at 20 μg/mL (inhibition ratio, %)

| **Compound** | **A549** | **BT-549** | **HeLa** | **HepG2** | **MCF-7** | **THP-1** |
| --- | --- | --- | --- | --- | --- | --- |
| (+)**-1** | 26.32% | −12.12% | 17.52% | 2.69% | −6.29% | −19.30% |
| (−)**-1** | 17.92% | −19.62% | 23.67% | 13.11% | −1.39% | −11.98% |
| **2** | 30.65% | 60.23% | 48.69% | 8.72% | 19.68% | 56.32% |
| **3** | 26.30% | 42.33% | 36.66% | 58.81% | 20.56% | 26.11% |
| **4** | −2.36% | 19.26% | 15.99% | 40.12% | −21.96% | 30.69% |
| **5** | 0.35% | 24.68% | −13.02% | 36.23% | −5.47% | −3.19% |
| **6** | −20.31% | 39.88% | 19.98% | 20.54% | 49.35% | 7.69% |
| **7** | 52.34% | 41.25% | 24.68% | −6.28% | 42.62% | 10.88% |
| Epirubicin^a^ | 92.57% | 90.46% | 90.20% | 96.13% | 87.65% | 82.56% |

^a^Positive control.

**Table S2.** Antifungal activity of compounds **1**–**7** (IC_50_, μg/mL)

| **Compound** | ***Alternaria solani*** | ***Botrytis cinerea*** | ***Fusarium oxysporum*** | ***Valsa mali*** |
| --- | --- | --- | --- | --- |
| (+)**-1** | ˃ 50 | 39.9 ± 0.9 | ˃ 50 | ˃ 50 |
| (−)**-1** | ˃ 50 | ˃ 50 | ˃ 50 | 40.2 ± 1.1 |
| **2** | 42.5 | 13.6 ± 0.5 | ˃ 50 | ˃ 50 |
| **3** | ˃ 50 | 29.6 ± 1.2 | 46.6 ± 1.5 | ˃ 50 |
| **4** | ˃ 50 | ˃ 50 | ˃ 50 | ˃ 50 |
| **5** | ˃ 50 | ˃ 50 | 35.6 ± 0.7 | 29.2 ± 1.0 |
| **6** | ˃ 50 | ˃ 50 | ˃ 50 | ˃ 50 |
| **7** | ˃ 50 | ˃ 50 | ˃ 50 | ˃ 50 |
| Carbendazim^a^ | 20.6 ± 0.9 | 19.2 ± 1.2 | 12.3 ± 0.6 | 9.9 ± 0.2 |

^a^Positive control.
